# Key Technologies of Physical and Virtual Test Rig for Railway Freight Car body

**DOI:** 10.3390/ma15155439

**Published:** 2022-08-08

**Authors:** Shangchao Zhao, Xiangwei Li, Dongpo Wang, Wenquan Li

**Affiliations:** 1 School of Materials Science and Engineering, Tianjin University, Tianjin 300350, China; 2 CRRC Qiqihar Rolling Stock Co., Ltd., Qiqihar 161000, China

**Keywords:** railway freight car, fatigue and vibration test rig, virtual test

## Abstract

On the one hand, considering that the traditional fatigue method of railway freight cars is based on damage as a parameter, the influence of stress waveform cannot be considered. On the other hand, physical experiments have the characteristics of lag, long period, and high cost. The full-scale physical test and virtual test of car body are carried out. First of all, the data processing method of small deletion and the inverse problem load acquisition method based on data to data are proposed. Secondly, the dynamic stress calculation method with the bench as the boundary is proposed. Finally, taking the obtained load as the input of the physical and virtual bench, a new fatigue test method for simulating the running attitude of the car body line is completed. The acceleration RMS error of the C70E gondola body is less than 6%, the stress RMS is less than 13%, and the equivalent mileage is 3.125 million highway test results show that the car meets the life requirements of the car body. The inverse problem analysis results of virtual and physical tests are basically consistent, and the study of this method provides a basis for improving the fatigue reliability of freight car bodies.

## 1. Introduction

With the development of railway technology, the load of the freight car body has changed, leading to fatigue failure of some railway freight products, e.g., cracks in the side column cushion plate of C80B coal gondola, cracks in the upper core plate of C80 aluminum alloy car, etc. These problems have caused great economic losses to manufacturing enterprises [1]. Considering these problems, in order to effectively support the fatigue reliability evaluation of railway freight cars, the fatigue and vibration test rig of railway freight cars of China Railway Qiqihar Rolling Stock Co., Ltd. was completed and put into use in 2012 by the TTCI (Transportation Technology Center) [2]. The fatigue test rig provides equipment for the research and development of heavy-duty freight car products [3,4].

The railway freight car is composed of car body, bogie, hook, etc. Usually, the external excitation is loaded on the wheel to obtain the dynamic response of the car body [5,6,7,8]. When considering the fatigue test of the car body, because the fatigue loading of the bogie is obviously higher than that of the car body, if the load is applied on the wheelset, the bogie will fail earlier than the car body, which cannot reflect the fatigue performance of the car body. Therefore, the full-scale car body fatigue test needs to separate the car body for the testing.

**Figure 1:**
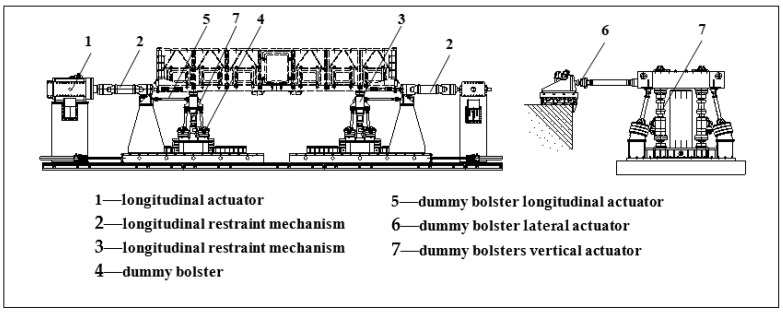
Full-size body fatigue test rig.

The fatigue test rig are mainly consisted in mechanical system, electro-control and safety monitoring system, test data collecting and processing system and related infrastructure. Loads can be applied by vertical, lateral, longitudinal, and coupling force actuators. The fatigue test rig is shown in Figure 1. The test rig is adjustable to accommodate railway gauges ranging from 1000 to 1676 mm, and car body with overall length up to 27,000 mm and of axle load up to 40 t [9]. The parameters of the actuator are shown in Table 1.

During the test, the car body for the test was supported by two dummy bolsters via center plates and side bearers. The static load of car body and loads can be supported by air springs under dummy bolsters. Dynamic loads are applied to the car body by four sets of vertical hydraulic actuators under dummy bolsters. Two longitudinal actuators are attached to each dummy bolster along the longitudinal line of the car body to balance the uneven coupling force and brake force at each end of car body and to simulate bogie rotation upon the center plate with respect to the car body and resultant rotation moment. At one end of the test rig, there is a single longitudinal coupler actuator connecting with car body to simulate coupler force. At the other end of car body, a longitudinal load application bar was fitted to connect car body end to center beam of longitudinal load application frame to counter coupler force. Thus, the tested car body can move to reproduce running in revenue service and to have dynamic response nearly the same as from revenue lines.

In order to improve the application of full-size car body fatigue test rig, physical simulation and virtual simulation experiments are carried out on the basis of the existing research results. This work aims to solve the following problems: (1) how to select the appropriate car body response as the target to obtain the equivalent load; and how to carry out virtual simulation and set up a digital model to simulate the physical test to reduce the high cost, and to overcome the limitation of physical test, such as the limited number of measuring points and so on. Through the combination of virtual and physical tests, we can provide a reference for product reliability design.

## 2. Full-Scale Fatigue Test Method

Due to the continuous dynamic testing of line operation, there are large amounts of data and many cycles of small loads. The design life of railway freight cars is usually 25 years. In order to quickly examine the fatigue reliability of the car body, it is necessary to accelerate the current approach via data compression processing and then carry out the line simulation test.

### 2.1. Data Compression

**Figure 2:**
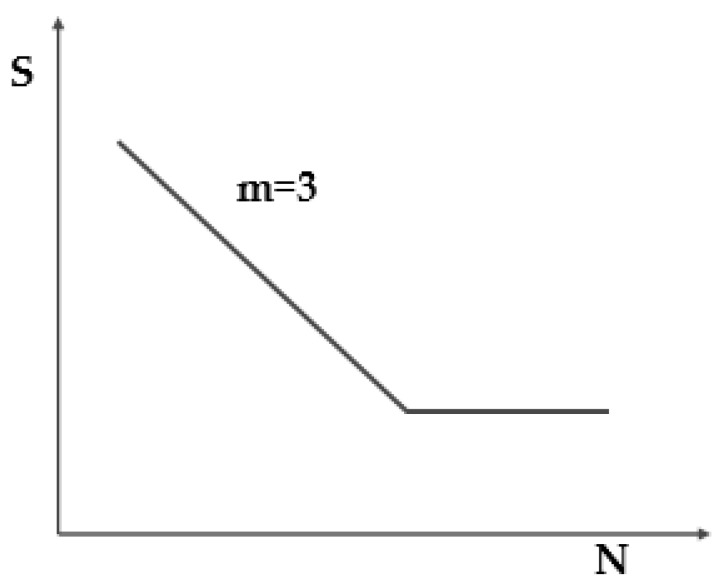
Schematic diagram of S-N curve.

**Figure 3:**
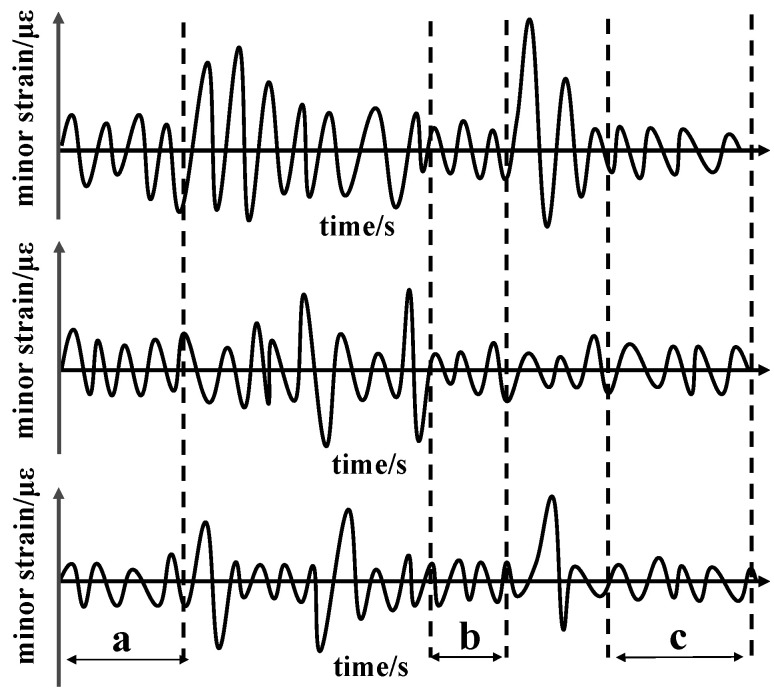
Correspondence of waveforms in the time domain curve.

Existing accelerated test methods include increasing the load frequency method, linear strengthening spectrum method, and deleting the small amount method [9]. The method of increasing the loading frequency has higher requirement in terms of test equipment. Due to the heavy load of the rolling stock, the hydraulic system of the rolling stock fatigue test rig has limited improvement in frequency. The linear reinforced spectrum method has a certain deviation from the actual measurement data and affects the test accuracy. Increasing the loading frequency and amplitude will change the loaded vibration state of the vehicle body and will not reflect the actual loading state of the rolling stock. These two methods are not suitable for the fatigue test of the car body. At present, the small amount of equal amplitude value or the non-equal amplitude value was deleted according to the measured data of the line. Statistical analysis of the load (or stress), the specified setting domain value, and deleting the fatigue damage are very important. Small load (or stress) events were deleted, thereby compressing test data to achieve accelerated fatigue testing. The main steps of the compression method used are as follows: (1) Rainflow count statistics are performed on the measured time domain signals after preprocessing, such as removing invalid signals, de-zeroing, and filtering. (2) According to the different S–N curves of each measuring point, as shown in Figure 2 [10,11,12], calculate the total damage after the rain flow statistics and delete the small vibration waveform without damage or minimal damage according to the test period. Considering the loading time, cost and other issues, the threshold value should be selected as scientific and reasonable as possible, and the damage equivalent principle should be fully followed. (3) When the non-damage small vibration waveform of a certain waveform is deleted, the mutual phase relationship between the wave forms was maintained, and the large vibration wave forms in the other channel wave forms are not deleted. As shown in Figure 3, the time domain wave forms of the three channels have a phase difference between the largescale vibration wave and stress wave forms. The vibration should be retained, so only the three measuring points can be deleted at the same time as the small vibration waveform, as shown in areas a–c, which can ensure the phase is unchanged after the cutting and retain the required large amplitude data. The crack propagation law can be considered in the non-equal amplitude domain data compression method. By setting the compression domain event to a higher compression domain value than the pull-up load event, the compression ratio of the data is increased, and the test efficiency is improved.

### 2.2. Simulation Test Method

After the compressed signal is obtained, the vertical and lateral acceleration are taken as the simulation target. Firstly, the signal composed of white noise and pink noise is excited in the test system, and the corresponding system response is obtained. The frequency response function [H(ω)] of the bench test system is calculated by input and output [10].

There are a total of 6 actuators in the vertical and lateral direction of the car body fatigue test bench. The displacement input white noise signal of the actuator cylinder can be expressed as $U(u_{1},u_{2},u_{3},u_{4},u_{5},u_{6})$. The acceleration response signal of pillow, or car body can be expressed as $Y(y_{1},y_{2}\ldots ,y_{n})$. For this multi-input and multi-output case, the frequency response function matrix of input and output is expressed as:

(1) $${\left\{Y(\omega )\right\}}_{n\times 1}={\left[H(\omega )\right]}_{n\times 6}{\left\{U(\omega )\right\}}_{6\times 1}$$

In the formula, Y(ω) is the Fourier function of response, U(ω) is the Fourier transform of input, and H(ω) is the frequency response function.

According to the definition of self-spectrum and cross-spectrum of input and output, Formula (1) can be expressed as:

(2) $${\left[H(\omega )\right]}_{n\times 6}={\left[G_{yu}\right]}_{n\times 6}/{\left[G_{uu}\right]}_{6\times 6}$$

In the formula, $G_{yu}$ is the mutual power spectrum of the input signal and the output signal, and $G_{uu}$ is the self-power spectrum of the input signal.

The inverse of the frequency response function is calculated, and the initial driving signal of the system is obtained by multiplying the line target and the inverse matrix of the frequency response function.

In order to avoid the system response obtained by the first drive signal greatly exceeding the target response signal, thus causing damage to the test system, and in order to prevent the iterative process from diverging, a weighting coefficient α is added to obtain the initial drive signal matrix and calculated in the frequency domain.

(3) $$U_{1}\left(j\omega \right)=H^{-1}\left(j\omega \right)Y_{1}\left(j\omega \right)\alpha$$

where $U_{1}(j\omega )$ is the Fourier transform of the initial driving signal matrix, the matrix $U_{1}(t)$ can be obtained by the inverse Fourier transform, $Y_{1}(j\omega )$ is the Fourier transform of the target signal, and the weighting coefficient is between 0 and 1.

At the beginning of the first iteration of the system, the response value under the initial driving excitation condition is obtained, the error between the response value and the line target is calculated, and the next driving signal is corrected by calculation according to the tracking error:

(4) $$\Delta U\left(j\omega \right)=H^{-1}\left(j\omega \right)E\left(j\omega \right)\beta$$

(5) $$U_{new}\left(j\omega \right)=U_{old}\left(j\omega \right)+\Delta U\left(j\omega \right)$$

In the formula, ΔU(jω) is the update amount of the driving signal matrix in the iteration. E(jω) is the Fourier transform of tracking error $E\left(j\omega \right)=Y_{t}(t)-Y(t)$. $U_{old}\left(j\omega \right)$ and $U_{new}\left(j\omega \right)$ are the driving signal matrix before and after the update.

The iterative process is repeated until the weighted error between the response, and the line target is less than the expected value, ε is as small as possible, and the line target response is considered to be reproduced in the bench system.

(6) $$\varepsilon =\frac{RMS\left\{e_{i}\left(t\right)\right\}}{RMS\left\{y_{i}\left(t\right)\right\}}\times 100\%$$

**Figure 4:**
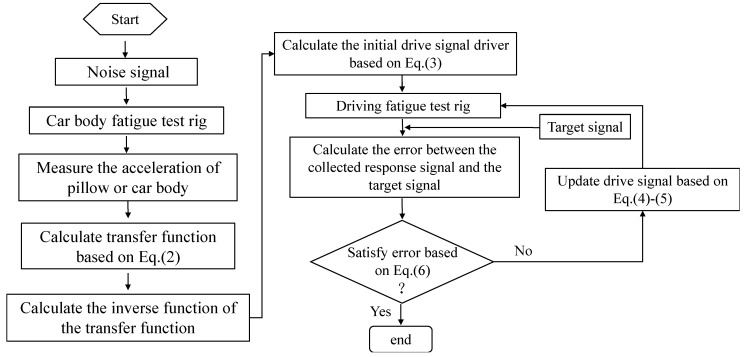
Simulation method of vertical and lateral vibration response of car body.

In the formula, $e\left(t\right)=y_{t}(t)-y(t)$ is the error signal and $y_{t}(t)$ is the target signal. According to the bench simulation theory, the circuit simulation process is shown in Figure 4.

### 2.3. Fatigue Test

The loads of vertical and lateral actuators are obtained by the TWR simulation test. The compressed coupler force as the driving force of the longitudinal actuator. Calculate equivalent service mileage from length of drive file for the tested car body. The cycles of drive file repeated shall be based on life expectancy. Before test, a warning or alarm for any abnormality shall be set up.

When equivalent mileage is achieved, car body conditions were checked. If there is no fatigue crack, wear, or deformation that may affect service of car, the tested car body is considered acceptable for required fatigue life. It is recommended to calculate accumulated damage to car body from data of monitoring points to evaluate fatigue life of monitoring points. If fatigue crack occurs and goes into thick plate, or a crack grows in different directions, it means that load bearing on the structure has changed. Recording the load application time period can be used to quantify car body fatigue life [9]. In special cases, tests may be stopped if a fatigue crack achieves specific length or if there is any wear or deformation as required by the customer. Record the load application time period and calculate it according to the equivalent mileage to evaluate fatigue life.

## 3. Fatigue Test of Gondola Car body

### 3.1. Revenue Service Test

**Figure 5:**
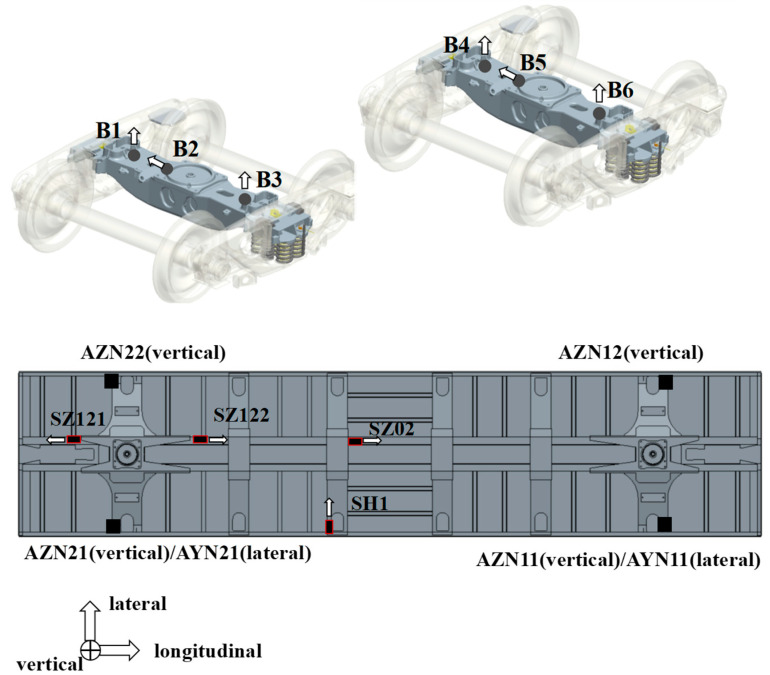
Location diagram of measuring point.

After the car body is isolated, without the connecting components of the bogie, the equivalent load of the oil cylinder on the bench is obtained by using the reverse calculation of the vibration state of the bolster or the vibration state of the car body. The equivalent excitation load can ensure that the test curve and fatigue damage of the critical load-bearing structure of the bench test and the line test is consistent. Therefore, in addition to the strain measuring points in the key parts of the car body, the following acceleration measuring points should also be tested: (1) Bolster acceleration measuring points. The vibration of these measuring points can fully reflect the vertical and transverse vibration of the pillow, and the measuring points are arranged on both sides of the side bearing. The lateral acceleration is arranged next to the center plate. (2) The measuring point of the acceleration of the car body should be arranged in the part of the larger stiffness of the car body, which can reflect the vibration modes, such as roll, torsion, and first-order vertical bending of the car body, and the measuring point should be arranged on the pillow beam of the car body, as shown in Figure 5.

**Figure 6:**
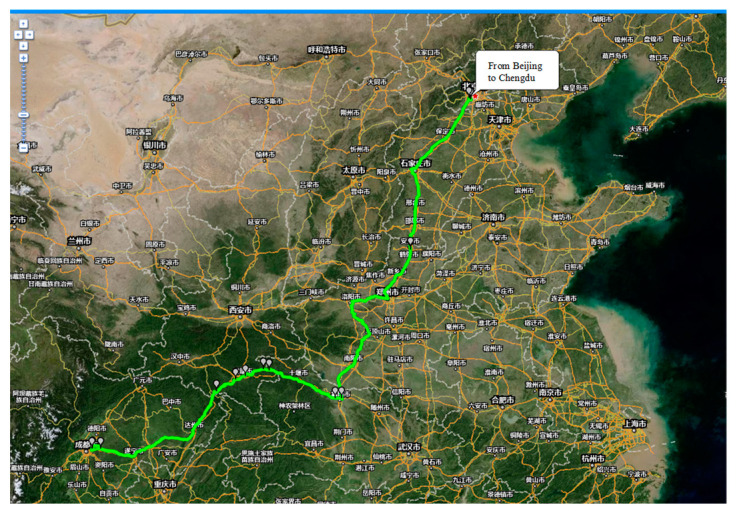
Schematic diagram of test circuit.

The test line is from Beijing to Chengdu, as shown in Figure 6. The total length of the test data is 456 h. After processing the measured original data, such as invalid data deletion, a group of effective data signals which can be used to simulate the vibration state and stress state of the tested vehicle are obtained. The effective signal length of the original data from Beijing to Chengdu after pre-processing is 44.1 h.

### 3.2. Data Processing

In order to improve the test efficiency, by identifying the sections with less fatigue damage in all the test data of the car body, setting the threshold to delete the small load events with less fatigue damage synchronously, and then taking the compressed data as the simulation target. The tested coupler force load is also involved in compression, and the bench longitudinal load simulation can be completed by loading the compressed load into the longitudinal actuator [9].

**Figure 7:**
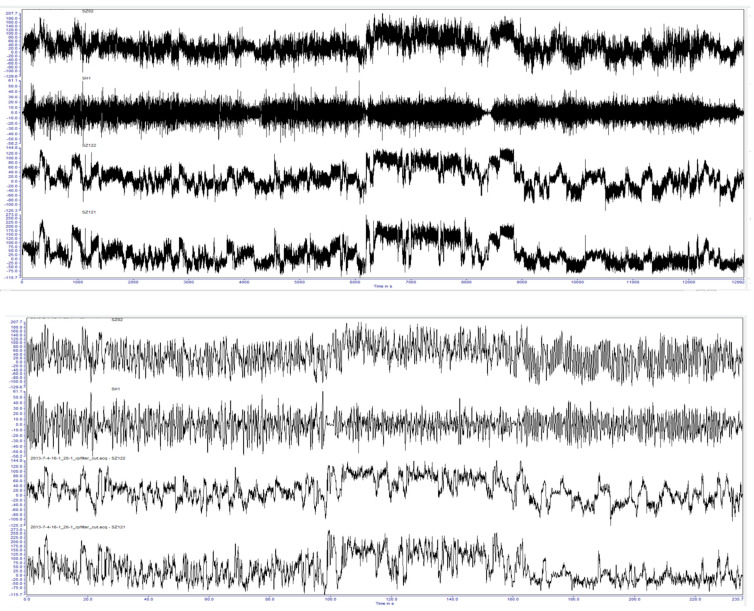
Comparison between before and after data compression.

The critical technology of data compression is to select the appropriate threshold. Take the 44-h dynamic response data compression of the Beijing-Chengdu line of the C70E available gondola as an example. As shown in Table 2, when the threshold of the 36 MPa N curve is selected, the compressed data is 0.176 h, the compression ratio is 0.4%, and the equivalent running time of the heavy vehicle of the car body fatigue test-rig is 270 h, if the test bench runs for 18 h a day. It takes 15 days to complete the car body fatigue test, 15 days to complete the car body fatigue test when the threshold is defined as 23 MPa, and 30 days to complete the car body fatigue test when the threshold is defined as 29 MPa. In order to take into account the influence of test efficiency and small damage data, the threshold of the S-N curve 29 MPa is defined as the most reasonable. Considering that the small load can also cause damage, after completing the conventional test, the number of cycles can be appropriately supplemented to do as similar damage as possible. The schematic diagram of data compression is shown in Figure 7.

### 3.3. Results of Different Simulation Targets

The measured acceleration data of the compressed line are selected as the simulation targets, including six accelerations of the bolster and car body pillow beam acceleration. The frequency response function from the actuator to the dummy bolster and the frequency response function from the actuator to the pillow beam of the car body is identified. Then, the line state is simulated by the control iterative algorithm, and the position of the simulation target is shown in Figure 5.

**Figure 8:**
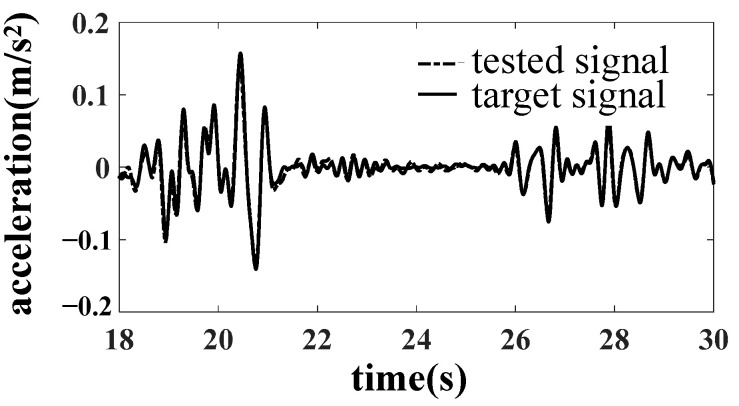
Comparison of B1 acceleration result.

**Figure 9:**
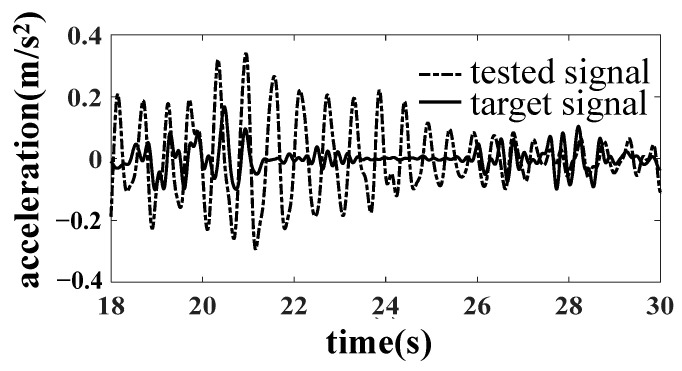
Comparison of AZN11 acceleration result.

**Figure 10:**
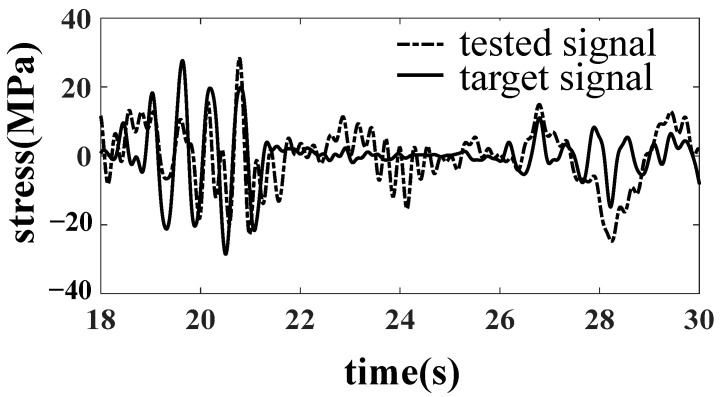
Comparison of SH1 stress result.

In the simulation, it is found that when taking the six accelerations of the bolster as the target, there is no nonlinear factor between the dummy bolster and the actuator. The linearity of the frequency response function from the actuator to the simulated pillow is high, so it is easy to complete the simulation by an iterative method. When the dummy bolster is transferred to the car body through the core plate and side bearing, the simulation effect of the car body is not very ideal because the core plate and side bearing have contact nonlinearity, especially the clearance of the core plate. As shown in Figure 8, the B1 acceleration of the bolster is well simulated. However, as shown in Figure 9 and Figure 10, the simulation results of the vertical acceleration AZN11 of the car body and the strain SH1 of the large crossbeam of the car body are poor.

**Figure 11:**
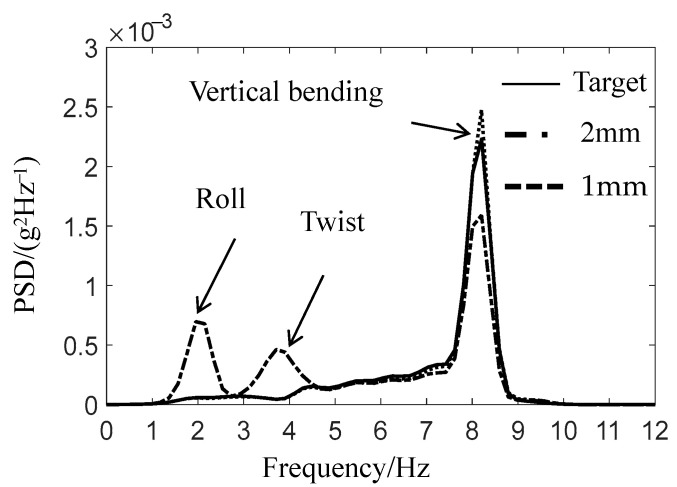
Comparison of AZN11 acceleration results before and after the improvement of core disc gap.

When taking the acceleration of the car body pillow beam as the simulation target, the actual simulation effect is not very ideal, and the car body dynamic magnification occurs [13,14]. After detailed analysis, it is found that the actual circular center disc gap is 2 mm, and the center disc gap can easily arouse the car body roll, torsion, and vertical first-order bending mode. When the simulation target is moved up to the car body, the problem of the center disc gap must be solved first, and the gap between the upper and lower center disc of the gondola should be reduced from the original 2 mm to 1 mm. The PSD analysis results of the comparison between the simulated car body response and the target signal before and after the reduction of the core disc gap is shown in Figure 11. The test results show that the careful disc clearance can effectively restrain the roll motion and torsional vibration mode of the car body, significantly reduce the influence of nonlinearity, and ensure the accuracy of line simulation.

**Figure 12:**
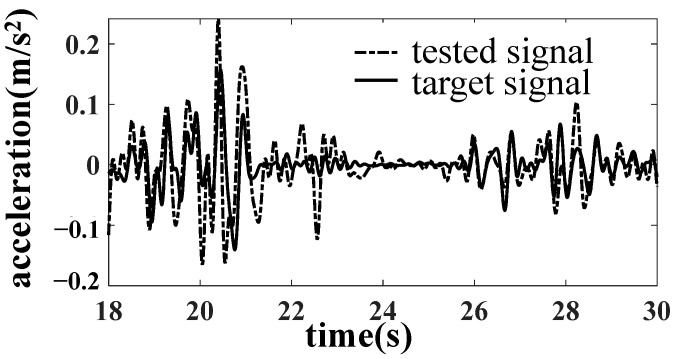
Comparison of B1 acceleration results.

**Figure 13:**
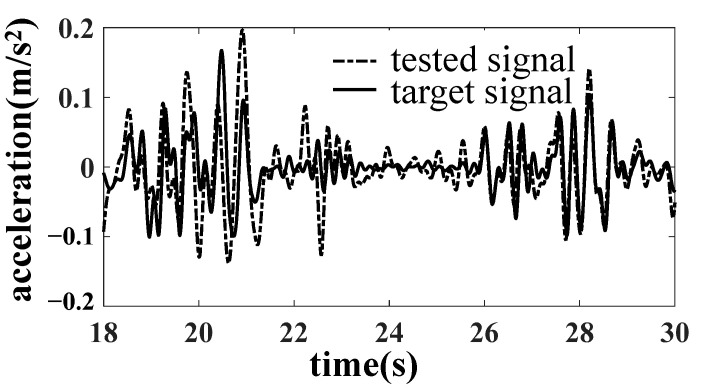
Comparison of AZN11 acceleration results.

**Figure 14:**
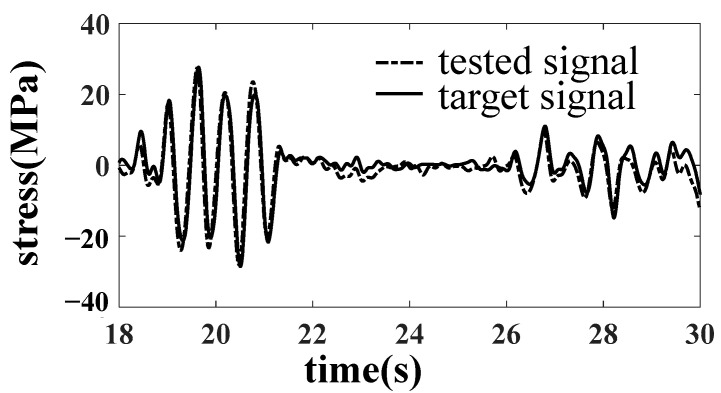
Comparison of SH1 stress results.

After reducing the gap of the core disc, the line simulation is carried out with the acceleration of the car body as the simulation target. The results are improved obviously, as shown in Figure 12, Figure 13 and Figure 14.

A drive file for car body fatigue test was created after many iterations. Response signals were compared with target signals for the calibration of drive file. Error of acceleration RMS is less than 6%, and error of stress RMS is less than 13%. The requirement of accuracy was verified.

### 3.4. Fatigue Test Evaluation

**Figure 15:**
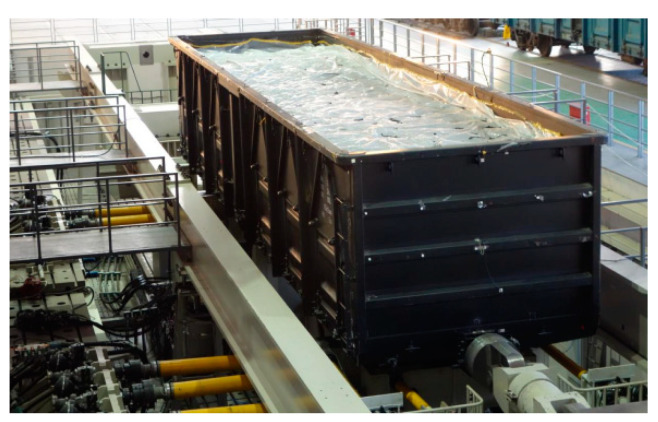
Fatigue test of C70E car body.

**Figure 16:**
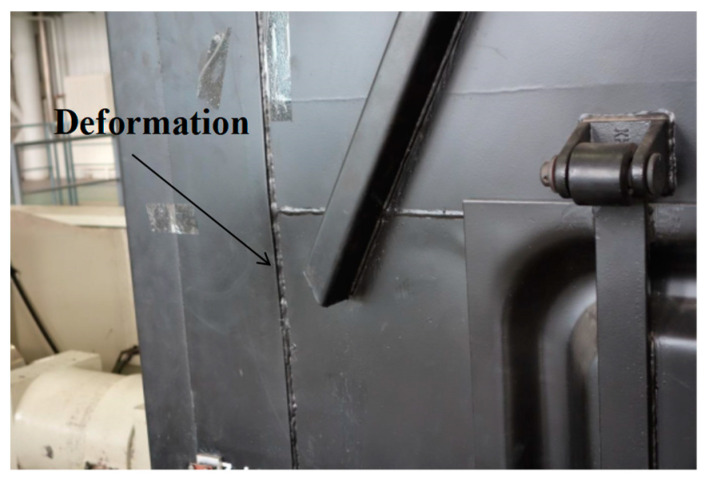
Deformation of car body after fatigue test.

The drive file was repeated 734 cycles to achieve accumulated test time of 499.1 h and equivalent total mileage of 3.142 million kilometers, as show in Figure 15. After the test, the car body was inspected. There was deformation on top chord and ends of side wall plate, as show in Figure 16. The deformation measured on the top chord is around 19.5 mm, within permissible tolerance according to regulations for the maintenance of railway wagons [15,16]. Critical welds and load bearing areas were also checked, and no fatigue crack was found. The car body is in good condition [17]. There was some deformation in the car body, as show in Figure 16. Based on response signals from test points, fatigue damages of some test points were calculated. The accumulated damage at flange plate of center sill outside center plate at A end of car body is 0.416, and at flange plate of center sill inside center plate at the end of car body is 0.307. Damages at other locations are very small and, therefore, were not evaluated [18]. The effectiveness of the key technologies in the fatigue test described above has been proven by the test results [19], also demonstrating that the method of accelerated fatigue testing based on service line test data was reasonable and appropriate to the evaluation of fatigue life.

The fatigue test of the car body was carried out to verify the rationality of the method. The car body fatigue test results reflect the weak parts of the car body and provide a force guarantee for improving the design quality of the car body.

In the simulation-assisted physical test analysis, it was found that the dynamic vertical and transverse cylinder dynamic and longitudinal coupler forces obtained from the line simulation test cannot be used in the traditional quasi-static analysis method. In order to solve the problem of the use of the bench load and make the physical test and simulation analysis complement and guide each other, it is necessary to carry out the simulation analysis method corresponding to the bench.

## 4. Virtual Test Rig

The modeling of the test rig system is carried out based on the rigid-flexible coupling multi-body dynamics theory. The test rig system consists of the mechanism and the car body. The test rig has the characteristic of large stiffness, so it is built into a rigid body. The stress of the car body is composed of elastic modes, so it is considered as a flexible body [16,17,18].

### 4.1. Digital Model of Test Rig

**Figure 17:**
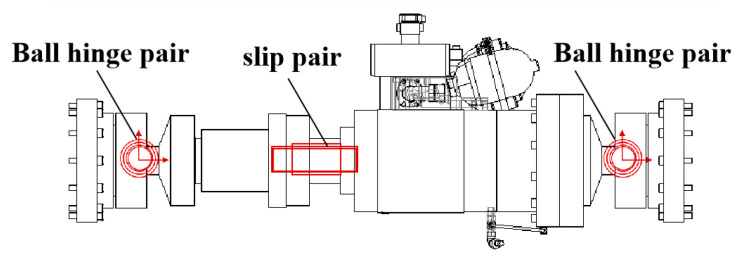
Schematic diagram of actuator.

The vertical, lateral, and longitudinal loading systems of the test rig are mainly composed of actuators and load transfer components. The actuator part is composed of two moving parts: the ball hinge and the oil cylinder. The ball hinge contains lubricating oil, and the actuator contains hydraulic oil, so the effect of friction cannot be considered in the modeling. The ball hinge is modeled according to the ball hinge pair, and the actuator is modeled according to the slip pair, as show in Figure 17. The modeling method of other actuators is consistent with that of vertical actuators.

The center plate and side bearing above the analog pillow are used to connect the car body, and the side and lower parts have connecting seats for connecting the actuator. The simulation pillow is the core component of the motion relationship, and the connectors on it must ensure the position accuracy. It is suggested to comprehensively consider the drawing size and the actual measurement results for modeling.

The side bearing on the dummy bolster is the constant contact side bearing, the vertical stiffness 2200 N/mm and the longitudinal stiffness 3500 N/mm. In the heavy vehicle condition, the distance between the upper surface of the side bearing and the roller clearance is 3~5 mm, so the side bearing stiffness should be a function of the roller clearance. When the assembly zero points to the compression 4 mm, the side bearing stiffness is 2200 N/mm, and when the compression continues, the stiffness becomes maximum due to the contact of the rollers, so the value in this paper is 400,000 N/mm.

The platform cannot simulate the running state of the curve, so the center plate on the pillow mainly completes the vertical and lateral load transfer and does not need to consider the rotary resistance. In the course of the test, considering the influence of the separation of the core plate, the core plate model is simplified to a four-point unilateral spring with large stiffness.

### 4.2. Car Body Simulation Model

The study of C70E opens as the research object. How to deal with the relationship between bulk and gondola car body is the key factor to ensuring the accuracy of the model. It is considered in reference [19] that the compressive yield strength of bulk materials is much larger than that of tensile yield strength, and the particles will expand when shearing, and the commonly used von-Mises yield criterion is no longer applicable. D-P model can be used to simulate the deformation and stress state of bulk more accurately. The calculation results show that the modal order of rigid body and elastic body are consistent, and the results of first-order vertical bending and first-order transverse bending are consistent. However, because the bulk cannot reflect the characteristics of motion lag, there is a certain error in roll and torsion frequency. The modal tests of the C70E gondola under different boundary conditions and different bulk loading conditions are carried out in reference [20,21]. The results show that the solid and loose state of bulk particles has little influence on the rigid body and elastomer frequency of the car body. Bulk cargo mainly affects the side roll mode and sidewall vibration of the car body.

**Figure 18:**
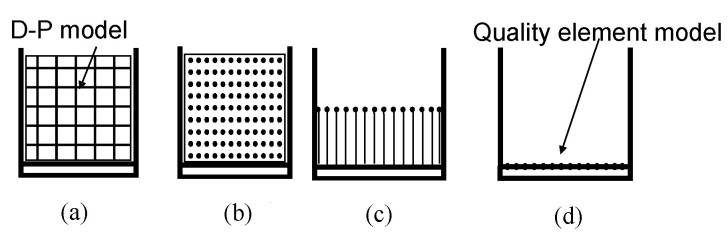
Modeling process of car body. (**a**) Solid modeling using D-P element; (**b**) Discrete into mass element; (**c**) Establish a mass unit at the center of mass; (**d**) Attach the mass element to the floor.

**Figure 19:**
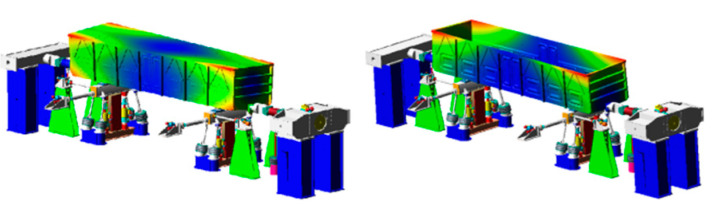
Simulation model of the rig.

Given the above analysis, the simplified process of the bulk body is designed as shown in Figure 18. On the one hand, the fatigue life of the weld structure of the car body underframe determines the fatigue life of the car body, while the sidewall has little influence on the service life of the car body. On the other hand, for the gondola, the rigid body modes such as roll contribute less to the stress of the car body, while the frequency of the elastic body makes a more significant contribution to the stress. Therefore, the bulk body is modeled by the (a) D-P model and (d) quality element model. After the modeling is completed, the flexible body is generated by the static interface method, and the assembled model is shown in Figure 19.

The damping ratio of the C70E gondola is based on the gondola modal test results, and the part exceeding the test frequency is set at 1%. After completing the system modeling, the core plate stiffness of the simulation model is modified based on the modal test results, and the properties of the modified C70E gondola simulation test system and the physical test system are the same.

**Figure 20:**
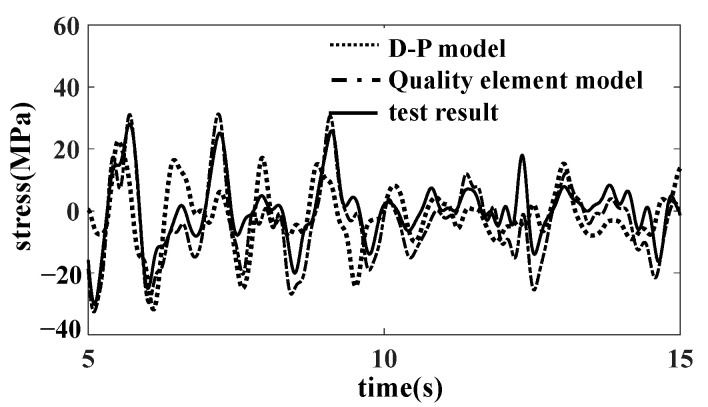
Comparison of SH1 results.

**Figure 21:**
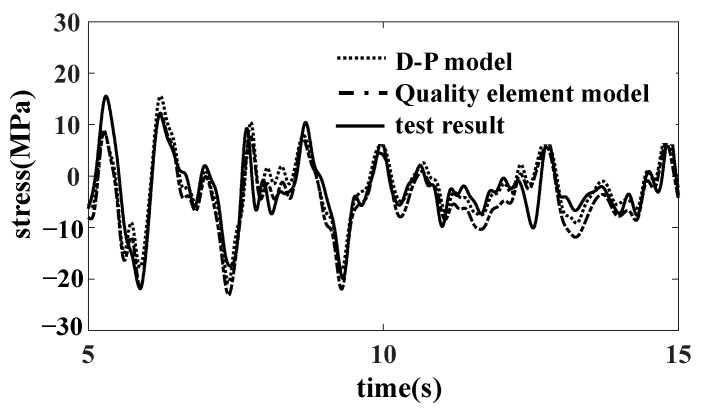
Comparison of SZ02 results.

The comparison results select the SH1 measuring point (affected by vertical and lateral loads) and the SZ02 measuring point (mainly affected by the longitudinal and vertical loads of the car body). The comparison results of the time domain curves of the measuring points SH1 and SZ02 are shown in Figure 20 and Figure 21. The damage results of the strain measuring points are shown in Table 3. The results of the quality element model are in good agreement with the experimental results, and there are some differences between the results of the D-P model and the experimental results. The quality element model is more convenient than the bulk model and better agrees with the test results.

## 5. Simulation Test of Virtual Test Rig

**Figure 22:**
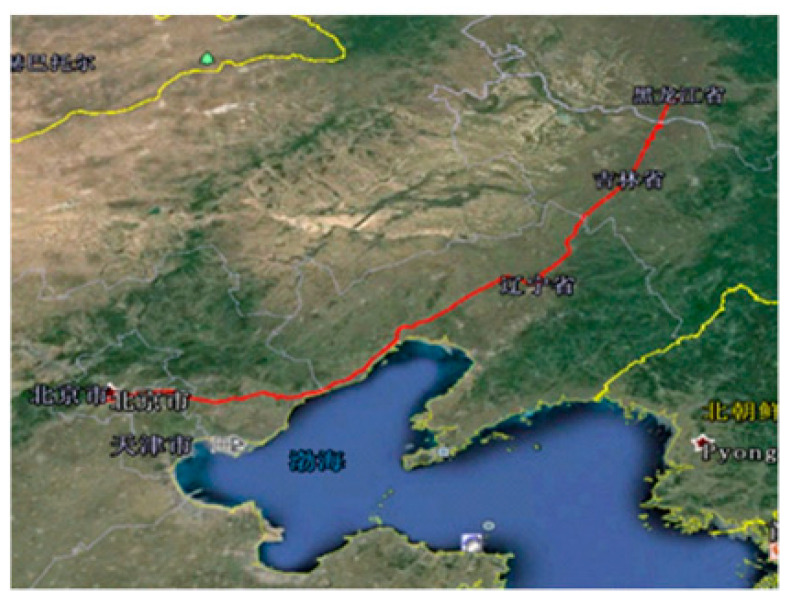
Schematic diagram of test circuit.

We also tested the line from Beijing to Harbin, as shown in Figure 22. Through data pre-processing, data compression, and driver file compilation, the target file from Beijing to Harbin was obtained.

Using virtual test model instead of physical test model to carry out simulation test. The C70E gondola is still taken as the research object.

**Figure 23:**
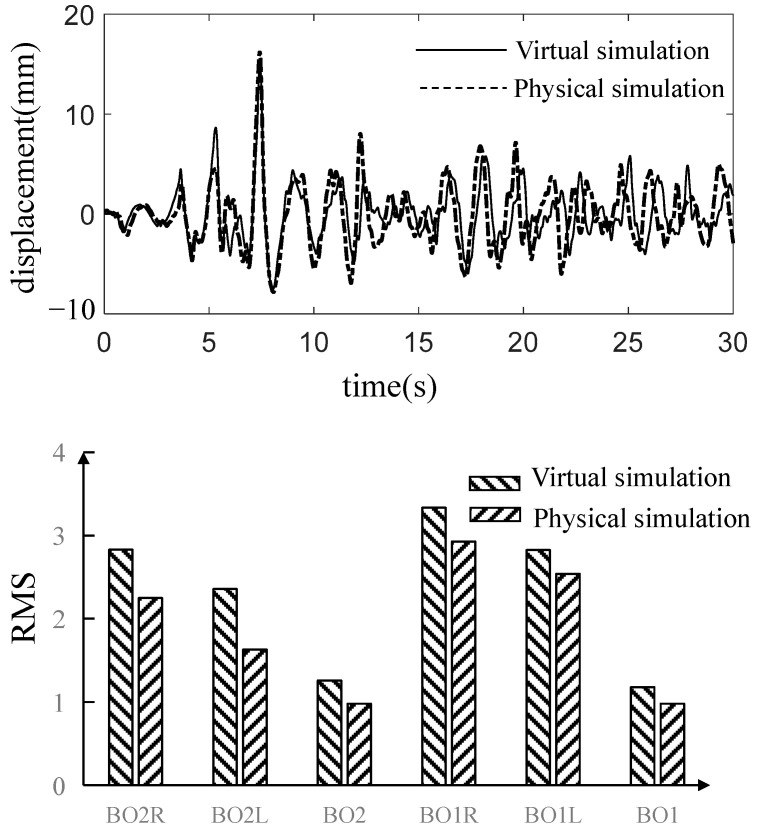
Comparison of simulation results between physical test and virtual test.

After nine iterations, the simulation accuracy of the acceleration of car body is not further improved. The displacement drive obtained by the virtual iteration is compared with that obtained by the real test bench. As shown in Figure 23, the results of the virtual circuit simulation test are basically the same as those of the physical test bench 10 s ago, and the two are different after 10 s. This is related to the coupling between the actuators of the suspension test-bed. However, it still has some similarity in regard to amplitude and trend, which shows the rationality of the virtual test.

The fatigue assessment of the driver obtained through this virtual bench shows that due to the good state of the line from Beijing to Harbin, the damage to the car body is very small, which meets the life requirements.

On the one hand, when there is no car body in the design stage, or it is difficult to bring back the existing vehicles to carry out the test, the virtual test method of railway freight cars becomes particularly important. On the other hand, when the physical test is carried out, and the scheme verification needs to be further carried out, the modified virtual test model is used to complete the verification, which can save the cost of a full-scale test.

## 6. Conclusions

(1) After the dynamic response test of the line, taking into account the influence of test efficiency and small load damage, the small load events with less fatigue damage are deleted synchronously by setting a reasonable threshold, so as to realize the acceleration of the simulation test.

(2) Because of the nonlinearity of the test bench, the iterative algorithm of system identification and remote parameter control should be adopted to carry out the simulation test of the railway freight car line. When the acceleration of the bolster is taken as the target, the simulation accuracy of the strain waveform of the car body is poor. Although the simulated damage of the bench can be consistent with the target damage of the line by adjusting the drive, the stress state of the car body has changed. When the acceleration of the car body is taken as the simulation target, the simulation position is placed on the pillow beam. At the same time, the gap between the core plate is reduced, and the nonlinear influence of the core plate is suppressed, the accuracy of strain simulation in the critical parts of the car body is improved. Taking the acceleration of the pillow beam as the target is more reasonable than that of the pillow acceleration.

(3) The virtual bench system established by the physical test bench can, on the one hand, obtain the equivalent load from the physical bench for fatigue simulation analysis, make up for the shortage of physical test points, and realize the life evaluation of the whole car body. On the other hand, it can carry out virtual circuit simulation tests and complete life evaluations in the case of a lack of test sample cars.

(4) Line simulation test technology plays an essential role in the research and development of railway freight cars. Through computer simulation technology, the line test data, indoor bench test, and product design stage reliability evaluation form a closed loop, which provides a guarantee for improving the reliability of products.

## Figures and Tables

**Table 1:** Parameters of test rig actuators.

Description	Max. Excitation Force (KN)	Qty.	Max. Travel (mm)	Working Frequency Range (Hz)
Coupling actuator	±3500	1	±200	0–30
Vertical actuator	±630	4	±75	0–30
Lateral actuator	±400	2	±75	0–30
Longitudinal actuator	±160	4	±75	0–30

**Table 2:** Efficiency of data compression.

Threshold of Compression	Before Compression/h	After Compression/h	Compression Ratio%	Time of Experiment/h
23	44.1	0.792	1.80%	1224
29		0.352	0.80%	540
36		0.176	0.40%	270

**Table 3:** Comparison of damage results of strain measuring points.

Measuring Point	Test Results	D-P Model	Quality Element Model
SH1	8.66 × 10^−8^	1.07 × 10^−9^	1.97 × 10^−9^
SZ02	4.63 × 10^−5^	6.62 × 10^−5^	5.98 × 10^−5^
SZ122	2.95 × 10^−5^	4.63 × 10^−5^	3.25 × 10^−5^
SZ121	5.73 × 10^−5^	5.13 × 10^−5^	5.90 × 10^−5^
